# Safranal-Standardized Saffron Extract Improves Metabolic, Cognitive, and Anxiolytic Outcomes in Aged Mice via Hypothalamic–Amygdalar Peptide Modulation

**DOI:** 10.3390/nu18020291

**Published:** 2026-01-16

**Authors:** Juan A. Navarro, Ana Gavito, Sonia Rivas, Alonso Rodríguez-Martín, Elena Baixeras, Juan Decara, Pedro J. Serrano-Castro, Yolanda Alfonso, Carlos Sanjuan, Antonia Serrano, Fernando Rodríguez de Fonseca

**Affiliations:** 1 Grupo de Neuropsicofarmacología, Instituto de Investigación Biomédica de Málaga y Plataforma en Nanomedicina-IBIMA Plataforma BIONAND, 29010 Málaga, Spain; juan_naga@hotmail.es (J.A.N.); analugavito@hotmail.com (A.G.); rivasmunozsonia@gmail.com (S.R.); alonsimiguel2002234@gmail.com (A.R.-M.); juan.decara@ibima.eu (J.D.); antonia.serrano@ibima.eu (A.S.); 2 Unidad Clínica de Neurología, Hospital Regional Universitario, Instituto de Investigación Biomédica de Málaga y Plataforma en Nanomedicina-IBIMA Plataforma BIONAND, 29010 Málaga, Spain; pedro.serrano.c@gmail.com; 3 Unidad Clínica de Salud Mental, Hospital Regional Universitario de Málaga, Instituto de Investigación Biomédica de Málaga y Plataforma en Nanomedicina-IBIMA Plataforma BIONAND, 29010 Málaga, Spain; 4 Departamento de Bioquímica, Biología Molecular e Inmunología, Facultad de Medicina, Universidad de Málaga, 29010 Málaga, Spain; ebaixeras@uma.es; 5 Andalusian Network for Clinical and Translational Research in Neurology [NEURO-RECA], 29001 Málaga, Spain; 6 Euronutra S.L., Calle Johannes Kepler, 3, 29590 Málaga, Spain; m.product@euronutra.com (Y.A.);

**Keywords:** aging, anxiety, appetite, brain, cognitive impairment, crocins, dementia, lipid metabolism, mice, saffron, safranal

## Abstract

*Background*: Population aging increases susceptibility to cognitive decline, anxiety, and metabolic dysregulation, yet safe and effective interventions remain limited. Saffron (*Crocus sativus* L.) has been traditionally used to enhance mood and cognition, and its main metabolites, crocins and safranal, exert neuroprotective, anxiolytic, and metabolic effects. However, variability in extract composition and frequent adulteration hinder reproducibility. *Objectives*: To clarify the efficacy of genuine saffron preparations in aging, we investigated a saffron extract standardized for safranal and crocin content (SSE). *Methods*: Safranal bioavailability was first characterized in rats, followed by an evaluation of behavioral, neuroendocrine, and metabolic outcomes after 35 days of oral SSE administration (25 or 200 mg/kg/day) in 25-month-old male C57BL/6 mice. Behavioral performance was assessed using open field and novel object recognition tests, while molecular analyses targeted neuropeptides in the hypothalamus and amygdala, hippocampal plasticity markers, cortical inflammatory proteins, and hepatic lipid metabolism genes. *Results*: SSE administration induced a rapid but transient increase in the plasma’s safranal, confirming its bioavailability. In aged mice, the low dose prevented age-related weight loss and modulated hepatic lipid metabolism, whereas the high dose reduced anxiety-like behavior and improved recognition memory. The anxiolytic effects are consistent with elevated hypothalamic *Npy*, an anxiolytic peptide, reduced amygdalar *Crh*, a key mediator of stress and anxiety, and decreased hypothalamic *Hcrt,* an arousal modulator. The improvement in memory is associated with modulation of the cortical and hippocampal inflammatory and endocannabinoid proteins involved in neural plasticity. *Conclusions*: These findings highlight content-standardized saffron extracts as a promising multi-target nutraceuticals for healthy aging.

## 1. Introduction

Population aging is associated with a surge in chronic and degenerative conditions, including cognitive decline, mood disorders, frailty, sensory derangement (vision or hearing loss), and metabolic disturbances, all of which undermine quality of life. Pharmacological strategies available for these conditions are often less effective and less safe in the elderly due to altered pharmacokinetics, polypharmacy, and multimorbidity. This therapeutic gap highlights the need for safe, multi-target interventions capable of acting across neurobehavioral, metabolic, and inflammatory processes. Ethnobotanicals and plant-derived bioactives, long employed in traditional medical systems, represent a particularly promising source of such interventions because of their pleiotropic actions and favorable safety profiles. Their multimodal nature aligns with the complex physiological disturbances of aging, providing opportunities for integrated support of mental and physical health [1,2,3,4].

**Figure 1:**
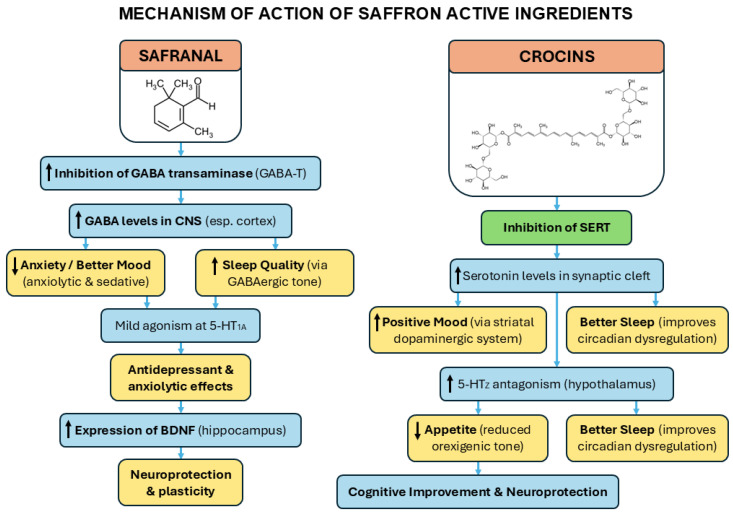
Mechanism of action of main saffron constituents: **left**, safranal; **right**, crocins.

Among these ethnobotanicals, saffron (*Crocus sativus* L.) stands out as one of the most ancient and valued medicinal spices, used for centuries in Persian, Ayurvedic, and Greco-Arabic traditions. Traditionally, saffron preparations were prescribed for mood elevation, cognitive support, pain relief, appetite modulation, and for the treatment of gastrointestinal and cardiovascular complaints [5,6]. These uses are now increasingly supported by modern pharmacology, which has identified several key bioactive constituents, including crocins (glycosylated carotenoids), safranal (a monoterpenoid aldehyde), and picrocrocin (a glycoside precursor of safranal). These compounds differ markedly in their physicochemical properties, tissue distribution, and pharmacokinetics, which together shape their biological activity [7,8,9,10]. Historical and contemporary evidence converge to suggest that saffron exerts health-promoting actions not through one isolated constituent, but through the interplay of its major metabolites. The complementary actions of crocins and safranal exemplify this synergy (see Figure 1). Crocins, hydrophilic molecules that are poorly brain-permeant, exert strong antioxidant and anti-inflammatory effects, protecting membranes, mitochondria, and synaptic structures [8,9,11]. Safranal, a lipophilic volatile compound, crosses the blood–brain barrier more readily and directly modulates neurotransmission, particularly GABAergic and monoaminergic pathways. Safranal has been shown to act as a positive allosteric modulator at GABA_A receptors (that is, potentiating inhibitory GABA transmission in a similar way to benzodiazepines), contributing to anxiolytic and sedative effects, while also influencing serotonergic signaling [12,13]. Crocins, in turn, reduce glutamate excitotoxicity, enhance antioxidant defenses, and stabilize mitochondrial function, possibly via the Nrf2 (nuclear factor erythroid 2-related factor 2) pathway, a key cellular defense system that regulates the expression of antioxidant and detoxifying enzymes) [14]. In addition, saffron extracts also modulate hypothalamic peptides such as arginine vasopressin and corticotropin-releasing hormone [15] to improve depressive symptoms in preclinical models. These peptides, together with NPY, are crucial signals for anxiety- and depressive-like responses [16,17]. Together, these molecules act in concert to improve memory, reduce depressive and anxious behaviors, and protect neurons against age-related insults. Clinical studies have shown that saffron extracts can perform comparably to standard antidepressants in mild-to-moderate depression and can enhance cognitive performance in individuals with mild cognitive impairment [18,19,20].

A major limitation in saffron research is the insufficient characterization of the extracts used. Most studies lack standardized methods to quantify key constituents, such as crocins and safranal, resulting in extracts without proper chemical authentication. Bioavailability data—particularly for safranal, a key chemical marker of genuine saffron products—are also scarce. Combined with inter-individual and interspecies variability in phytochemical metabolism, these gaps help explain the difficulty in reproducing preclinical findings with saffron extracts [4,6,7,10,19]. To further complicate the situation, the high value of saffron has also made it one of the most adulterated plant products worldwide. Market analyses reveal that between 20% and 30% of commercial saffron is adulterated, with rates as high as 60% in some regions [21]. Common fraud practices include substitution with cheaper plant materials, such as safflower (*Carthamus tinctorius*), calendula (*Calendula officinalis*), or turmeric (*Curcuma longa*); the addition of synthetic dyes such as tartrazine or ponceau-4R; moisture or weight enhancement through sugars, oils, or minerals; and mislabeling of low-quality saffron as premium batches. These practices not only reduce concentrations of bioactive compounds but also compromise safety and therapeutic efficacy. A further complication is that some adulterants, such as *Gardenia jasminoides* extracts or safflower, can provide crocins but not safranal, yielding extracts that superficially resemble saffron in color but lack critical neuroactive properties. Fraud therefore distorts both consumer confidence and the reproducibility of clinical and preclinical research [22,23]. To address these concerns, modern authentication tools, including spectroscopy, chromatography, high-resolution mass spectrometry, and artificial intelligence-enhanced infrared methods, are increasingly employed to detect adulteration with high accuracy [22,23,24].

The present study addressed the challenge of generating robust preclinical data by using a standardized saffron extract containing 2% safranal and 3% crocins, prepared through water extraction followed by chromatographic purification. We evaluated safranal’s bioavailability and the extract’s pharmacological actions by assessing behavioral outcomes—feeding behavior, locomotion, anxiety, and memory—and by analyzing brain molecular signatures associated with these responses (e.g., hypothalamic neuropeptides for feeding regulation, amygdalar neuropeptides for anxiety, and cortical/hippocampal neuroinflammation and endocannabinoid markers for cognition) after chronic administration. Aged mice were selected because standardized saffron represents an ethnobotanical therapeutic opportunity for the metabolic dysfunction, appetite changes, mood disturbances, and cognitive decline characteristic of aging [25].

## 2. Materials and Methods

### 2.1. Animals and Ethics Statement

All experiments were realized in compliance with the ARRIVE guidelines [26] and concordance with the European Communities Council Directives 2010/63/EU, Regulation (EC) n° 86/609/ECC (24 November 1986), and Spanish National and Regional Guidelines for Animal Experimentation (Real Decreto 53/2013). Experimental protocols were approved by The Local Ethical Committee for Animal Research of the University of Malaga (CEUMA n° 203-2023-A, 1 April 2024). Accordingly, all efforts were made to minimize animal suffering and to reduce the number of animals used. Potential confounders, such as the order of treatments and measurements, and animal/cage location, were minimized by standardizing the timing of treatments and measurements for all animals and ensuring consistent environmental conditions across cages. Additionally, cage locations were rotated periodically to prevent location bias. These measures were implemented to reduce the influence of external variables on the study outcomes.

For the safranal absorption study, adult male Wistar rats (Crl:WI(Han)) weighing 350 ± 20 g were used (Charles River Laboratories, Barcelona, Spain). For the chronic administration study, experiments were performed with 25-month-old male C57BL/6 mice (Charles River Laboratories, Barcelona, Spain). Animals were acquired at 1 year of age and remained undisturbed in our vivarium for an additional 13 months, caged in groups of 5. All animals were maintained under standard conditions (12/12 h light/dark cycle) with temperature (20 ± 2 °C) and humidity (40 ± 5%) control. Animals were fed a standard pellet diet (3.02 kcal/g) containing 30% kcal from protein, 55% kcal from carbohydrates, and 15% kcal from fat, purchased from Harlan Teklad (Madison, WI, USA). Water (with or without treatment) and food were available ad libitum.

### 2.2. Pharmacological Treatment with Safranal-Standardized Saffron Extract (SSE) and Monitorization of Safranal Plasma Levels

For all the experiments, we used a safranal-standardized saffron extract, SSE, with a consistent safranal content of 2% and a total crocins content of 3%, as measured by HPLC (see Supplementary Figure S1). The extract was generated by Euronutra S.L. (https://euronutra.com/, Málaga, Spain) using a water extraction and chromatographic separation procedure. Details of the composition and chromatographic analytical method used for the analysis of contents can be found in the Supplementary Materials. Doses of SSE selected for these preclinical studies were based on doses reported in saffron extract studies in humans [9,10,11,12,13,14,15,16,17,18], corrected for differences in metabolism and clearance, as described in Nair et al. [27]. The doses used in the present study were at least twenty-five times lower than the lethal dose in rats and a hundred times lower than the lethal dose in mice [28]. Wistar rats were used to check absorption of safranal, since this species can provide a sufficient plasma amount for bioanalytical methods, while aged mice from our colony were used for pharmacological evaluation of chronic oral administration of the extract on aging-related variables.

#### 2.2.1. Absorption of Safranal After Acute Oral Administration of SSE

Wistar rats received a single oral dose of 100 mg/kg SSE (equivalent to 2 mg/kg safranal) in 2 mL of water by gavage. Animals (n = 6 per time point) were sacrificed under anesthesia immediately after administration (time 0) and at 45, 90, and 180 min post-administration. Blood samples were collected and centrifuged to obtain plasma, which was stored at −80 °C until safranal concentration analysis.

##### Measurement of Plasma Levels of Safranal

Plasma safranal concentrations were monitored by the Medina Foundation (Parque Tecnológico de Las Ciencias de la Salud, Granada 18016, Spain). A quantitation method for safranal in rat plasma using liquid chromatography coupled with mass spectrometry (LC-MS/MS) was validated. The main objective was to establish a sensitive, precise, and reproducible procedure to detect this volatile and bioactive compound, derived from saffron, in complex biological matrices.

Analysis was carried out using an Agilent 1290 HPLC system (Agilent Technologies, Santa Clara, CA, USA) equipped with an Atlantis T3 column (2.1 × 5 mm, 3 µm), operated at 30 °C, with an injection volume of 5 µL. The mobile phase followed a gradient of acetonitrile and water acidified with 0.1% formic acid. The following two phases were used: phase A (water/acetonitrile 9:1) and phase B (acetonitrile/water 9:1), allowing efficient separation within a run time of 6.5 min. The autosampler temperature was maintained at 4 °C to preserve analyte stability.

Safranal was quantified by mass spectrometry operated in multiple reaction monitoring (MRM) mode, using an electrospray ionization (ESI) source in positive ion mode. The monitored transitions for safranal were m/z 151.0 → 123.0 and 151.0 → 81.1, with collision energies of 15 V and 23 V, respectively. Verapamil (m/z 455.3 → 165.3, CE: 37 V) served as the internal standard and was prepared at a final concentration of 150 ng/mL in acetonitrile. Stock solutions of safranal and verapamil were prepared in 100% DMSO at concentrations of 3 mg/mL and 2 mg/mL, respectively. These were used to generate a calibration curve of interference-free plasma, spanning concentrations from 156 ng/mL to 20,000 ng/mL. Calibration standards, quality control samples, and blanks were prepared by adding 2 µL of the appropriate solution to 50 µL of plasma.

Sample preparation involved adding 150 µL of chilled acetonitrile, containing the internal standard, to 50 µL of plasma. After vortex mixing for 1 min, the samples were centrifuged at 13,300 rpm for 15 min at 4 °C. A 160 µL aliquot of the supernatant was then collected for LC–MS/MS analysis.

Data were processed using Analyst software version 2.1.1, generating calibration curves based on the ratio of areas between the analyte and the internal standard. This ratio was used to determine safranal concentrations in biological samples with high precision and sensitivity, thereby validating a robust method for pharmacokinetic and toxicological studies in animal models.

#### 2.2.2. Study of SSE Efficacy Following Its Chronic Administration

A total of 25 male-aged C57BL/6 mice were used. Animals were acquired at 12 months of age and kept in the vivarium for additional 13 months. One animal assigned to the SSE dose of 25 mg/kg died before starting the experiment. For the treatment, they were caged individually and allocated as follows: a control group treated with water (n = 9) and two experimental groups receiving SSE in drinking water at doses of 25 mg/kg/day (n = 7) and 200 mg/kg/day (n = 8). The study lasted 35 days, beginning when the mice were approximately 25 months old and ending at 26 months of age. SSE treatment was administered daily via drinking water, which was newly prepared every other day and placed in opaque drinking bottles. On day 28 of treatment, the open field (OF) test was performed, followed by the novel object recognition (NOR) test. Behavioral tests were conducted by trained observers who were unaware of the experimental conditions. No software was used for the scoring of the open field test.

### 2.3. Behavioral Analyses

#### 2.3.1. Measurement of Food Intake and Body Weight

During the chronic administration study, body weight, food intake, and drink intake were recorded three times per week (Monday, Wednesday, and Friday) for each mouse. Intake values were calculated as the difference between the amount supplied and the amount remaining in each case.

#### 2.3.2. Open Field Test

This test assesses the animal’s tendency to explore a novel environment and is useful for detecting the anxiolytic or anxiogenic effects of the treatment. It was performed as described previously [29]. The test was conducted in an apparatus (40 × 40 cm, made of gray Plexiglas) divided into a central zone (8 × 8 cm) and a peripheral zone. The open field was illuminated using a ceiling halogen lamp that was regulated to yield 350 lux at the center of the field. Each mouse was placed in the center of the arena and observed for a 5 min period, during which horizontal activity (distance moved) and the percentage of time spent in the central zone (a common indicator associated with anxiety) were recorded.

#### 2.3.3. Novel Object Recognition Test

This test was selected for monitoring short-term memory. The test was conducted 1 h. after the open field and in the same apparatus used so the test was performed under familiar field conditions. The test was divided into two phases:

- Familiarization phase: 1 h after the habituation, two identical objects were placed in the upper and lower corners of the apparatus (with left–right positions counterbalanced), and mice were again placed in the field and allowed to explore both objects for 10 min.
- Test phase: 90 min after the familiarization phase, mice were returned to the arena, where one of the objects was replaced with a novel object differing in shape and size. Over the next 5 min, the time spent exploring each object was recorded.

During the test phase, the individual exploration time for each object (i.e., touching them with the nose or forepaws, analyzed observationally) and the total exploration time for both objects were recorded. The percentage of novelty preference (recognition index) was calculated using the following formula: (tNO*100)/(tFO + tNO), where tNO is the time that the animal spends exploring the novel object and tFO is the time spent exploring the familiar object. Preference for the novel object is interpreted as an indicator of intact short-term memory, leveraging the natural tendency of rodents to explore unfamiliar stimuli [29].

### 2.4. Tissue Sampling and Biochemical Procedures

#### 2.4.1. Sample Collection

Before sacrifice, animals were anesthetized by an intraperitoneal injection of sodium pentobarbital (50 mg/kg) administered five minutes prior to sacrifice, and blood was collected directly from the right atrium. Blood samples were centrifuged (2100× *g* for 10 min) to obtain plasma, which was stored at −80 °C for subsequent biochemical analysis of safranal. Brain and liver samples were rapidly excised, flash-frozen in dry ice, and stored at −80 °C until biochemical analysis. The total time from pentobarbital injection to sample freezing was less than 10 min, minimizing the impact of anesthesia on mRNA and protein expression.

For brain dissection, frozen brains were placed in a stainless-steel mouse brain matrix, and 2 mm-thick coronal sections were cut using razor blades. Based on Paxinos and Watson’s mouse brain atlas [30], the hypothalamus, amygdala, hippocampus, and prefrontal cortex were dissected.

#### 2.4.2. Real-Time PCR Analysis of Brain Areas and Liver Samples

Consumption of saffron preparations has been traditionally associated with changes in feeding behavior, mood, memory, and metabolism [7,8,9]. In order to establish the biological basis of SSE actions on the brain and liver, we selected a set of genes related to these pharmacological actions to be monitored by real-time PCR, as described below (see Supplementary Table S1 for primers used). Mouse liver and brain region sections were homogenized on ice, and total RNA was extracted from the tissue using the TRIzol^®^ method, according to the manufacturer’s instructions (Thermo Fisher Scientific, Waltham, MA, USA). RNA samples were then purified using the ReliaPrep™ RNA Clean-Up and Concentration System kit according to the manufacturer’s instructions (Promega, Madison, WI, USA; cat. no. Z1073). After purification, RNA samples were quantified using a NanoDrop™ ND-1000 spectrophotometer (Thermo Fisher Scientific, Waltham, MA, USA) to ensure A260/280 ratios between 1.8 and 2.0. Reverse transcription was performed using 1 µg of mRNA in a total reaction volume of 20 µL, using the qScript XLT cDNA SuperMix (Quantabio, Beverly, MA, USA).

##### Real-Time qPCR and Gene Expression Analysis

Real-time qPCR reactions were performed in a CFX Duet Real-Time PCR System (Bio-Rad, Hercules, CA, USA) for each cDNA template in a 10 µL reaction volume containing 4.5 µL of cDNA (previously diluted 1:100, corresponding to a total of 2.25 ng of cDNA per reaction) and 5.5 µL of PerfeCTa qPCR ToughMix (Quantabio, Beverly, MA, USA) containing 0.5 µL of the corresponding primer. All primers were obtained based on TaqMan^®^ Gene Expression Assays and the FAM™ dye label format (Thermo Fisher Scientific, Waltham, MA, USA). Supplementary Table S1 details the specific primers used for the amplification of each gene analyzed. Each reaction was run in duplicate. The cycling parameters were as follows: 50 °C for 2 min to degrade single- and double-stranded DNA containing dUTPs; 95 °C for 10 min to activate Taq DNA polymerase; followed by 44 cycles at 95 °C for 15 s for cDNA denaturation and 60 °C for 1 min for primer annealing and extension, during which fluorescence was acquired. A melting curve analysis confirmed that a single product was amplified. For relative quantification, the mean of the duplicates was used. Values obtained from each sample were normalized to *Actb* or *Gapdh* (used as reference genes), which did not vary significantly between groups. Gene expression in the control group treated with water (VEH) was arbitrarily set to one [31].

Using this technique, we analyzed the hypothalamic expression of the mRNA coding for peptides and receptors related to feeding regulation, including orexigenic and anorexigenic responses: *Npy* (Neuropeptide Y), *Cnr1* (Cannabinoid Receptor 1), *Agrp* (Agouti-Related Neuropeptide), *Mc4r* (Melanocortin 4 Receptor), *Pomc* (Proopiomelanocortin), and *Hcrt* (hypocretin/orexin). In the amygdala, we monitored the mRNA of expression of genes related to anxiety and stress responses, such as *Crh* (corticotropin-releasing hormone), *Crhr1* (corticotropin-releasing hormone receptor 1), *Crhr2* (corticotropin-releasing hormone receptor 2), *Npy* (Neuropeptide Y), *Nr3c1* (Nuclear Receptor Subfamily 3 Group C Member 1/Glucocorticoid Receptor), and *Nr3c2* (Nuclear Receptor Subfamily 3 Group C Member 2/Mineralocorticoid Receptor). In the hippocampus, we measured genes related to trophic factors, peptides, and receptors involved in neural plasticity and learning, such as *Bdnf* (Brain-Derived Neurotrophic Factor), *Ntrk2* (Neurotrophic Receptor Tyrosine Kinase 2), *Npy* (Neuropeptide Y), *Gria1* (Glutamate Ionotropic Receptor AMPA Type Subunit 1), *Gria2* (Glutamate Ionotropic Receptor AMPA Type Subunit 2), *Grin1* (Glutamate Ionotropic Receptor NMDA Type Subunit 1), *Grin2a* (Glutamate Ionotropic Receptor NMDA Type Subunit 2A), *Grin2b* (Glutamate Ionotropic Receptor NMDA Type Subunit 2B), and *Grm5* (Glutamate Metabotropic Receptor 5) gene expression. Finally, in the liver, since saffron extracts have also been attributed to modify weight gain and metabolism, we measured the mRNA expression of genes related to lipid metabolism, including *Stat3* (Signal Transducer and Activator of Transcription 3), *Acaca* (Acetyl-CoA Carboxylase Alpha), *Scd1* (Stearoyl-CoA Desaturase 1), *Acox1* (Acyl-CoA Oxidase 1), and *Cpt1a* (Carnitine Palmitoyltransferase 1A).

#### 2.4.3. Western Blot Analysis

We used the Western blot technique to measure the protein expression of the following: (a) inflammation-related proteins in the prefrontal cortex, including NF-κB (Nuclear Factor kappa-light-chain-enhancer of activated B cells), *p*-NF-κB (phosphorylated Nuclear Factor kappa-light-chain-enhancer of activated B cells), COX-2 (Cyclooxygenase-2), IKKβ (Inhibitor of Nuclear Factor kappa-B Kinase subunit beta), iNOS (Inducible Nitric Oxide Synthase), GFAP (Glial Fibrillary Acidic Protein), IBA1 (Ionized Calcium-Binding Adapter Molecule 1), and CB2 (Cannabinoid Receptor 2); and (b) endocannabinoid/paracannabinoid-related proteins in the hippocampus, including CB1 (Cannabinoid Receptor 1), DAGLα (Diacylglycerol Lipase Alpha), DAGLβ (Diacylglycerol Lipase Beta), MAGL (Monoacylglycerol Lipase), PPARα (Peroxisome Proliferator-Activated Receptor Alpha), NAPE-PLD (N-acyl Phosphatidylethanolamine-Specific Phospholipase D), FAAH (Fatty Acid Amide Hydrolase), CB2 (Cannabinoid Receptor 2), and GPR55 (G Protein-Coupled Receptor 55). Total protein from either the hippocampus or prefrontal cortex regions was extracted using ice-cold lysis buffer, as previously described [32]. Equivalent amounts of protein extract (30 μg) were separated on polyacrylamide gels: 4–12% (Bis-Tris) or 10% (Bis-Tris) Criterion XT Precast Gels (Bio-Rad, Hercules, CA, USA; cat. nos. 3450124 and 3450112, respectively), and electroblotted onto nitrocellulose membranes (Bio-Rad, Hercules, CA, USA; cat. no. 1620115). Membranes were blocked in TBS-T (50 mM Tris-HCl, pH 7.6; 200 mM NaCl; 0.1% Tween 20) containing 2% bovine serum albumin, fraction V (BSA; Roche, Basel, Switzerland) for 1 h at room temperature. Specific proteins were detected by overnight incubation at 4 °C with the corresponding primary antibodies (diluted in TBS-T containing 2% BSA). The specific primary antibodies used for protein expression analysis by Western blotting are listed in Supplementary Table S2. After extensive washing in TBS-T, HRP-conjugated anti-rabbit or anti-mouse secondary antibodies (Promega, Madison, WI, USA; cat. nos. W4011 and W4021, respectively), diluted 1:10,000 in TBS-T containing 2% BSA, were added for 1 h at room temperature. Following additional washes with TBS-T, specific proteins were visualized using the ECL™ Prime Western Blotting System (GE Healthcare, Chicago, IL, USA; cat. no. RPN2236), according to the manufacturer’s instructions. Images were captured using the ChemiDoc MP Imaging System (Bio-Rad, Hercules, CA, USA) [32].

After measuring phosphorylated proteins, the specific antibodies were removed from the membranes by incubation with a stripping buffer (2% SDS; 62.5 mM Tris-HCl, pH 6.8; 0.8% β-mercaptoethanol) for 30 min at 50 °C. Membranes were extensively washed in ultrapure water and then re-incubated with the corresponding antibody specific for the total protein. Quantification was performed using ImageJ software version 1.38 (Rasband, W.S., ImageJ, U.S. NIH, https://imagej.net/ij/, accessed on 13 August 2025). The signal intensity for total proteins was normalized to that of the corresponding control protein Adaptin band in the same blot. The phosphorylation state of a protein was expressed as the ratio of the signal obtained with the phospho-specific antibody to that obtained with the appropriate total protein antibody. The amount of the protein of interest in water-treated control samples (VEH) was arbitrarily set to 1.

### 2.5. Statistical Analyses

All data are expressed as mean ± standard error of the mean (SEM). Statistical analysis for PCR, Western blot, and multiplex analyses was conducted in GraphPad Prism, version 9 (GraphPad Software, Inc., La Jolla, CA, USA). The Shapiro–Wilk test was used to assess the normal distribution of data. Levene’s test was used to analyze the assumption of homogeneity of variance. Statistical analysis was undertaken for studies where each group size was, at least, n = 5. One-way analysis of variance (ANOVA) was assessed, followed by Dunnett’s post hoc comparison test of the two doses of *SSE* with the control group. The post hoc test was conducted only if F in one-way ANOVA or the interaction between factors in two ANOVA tests achieved a *p*-value of less than 0.05 and there was no statistically significant variance in homogeneity. Two-way ANOVA and Tukey’s test for multiple comparisons were performed for the time series of data (food intake, water intake, and body weight gain). The results were considered statistically significant at *p* ≤ 0.05. Non-significant (ns) results were above 0.05.

To address the potential issue of a Type-1 error arising from multiple outcome measures, power analysis and sample size estimation were performed before the initiation of the study. Effect sizes were estimated based on the previous literature to ensure adequate statistical power for the multitude of outcome measures. In this study design, the expected treatment effect corresponds to a mean difference of 35% relative to the control, with within-group variability set at 20% of the mean. This yields a standardized effect size of approximately 1.75. Based on a one-way ANOVA followed by Dunnett’s test (two-sided α = 0.05, familywise), the asymptotic calculation indicates a requirement of 7 subjects per group to achieve 80% power.

## 3. Results

### 3.1. Time-Course of Plasma Safranal Levels

**Figure 2:**
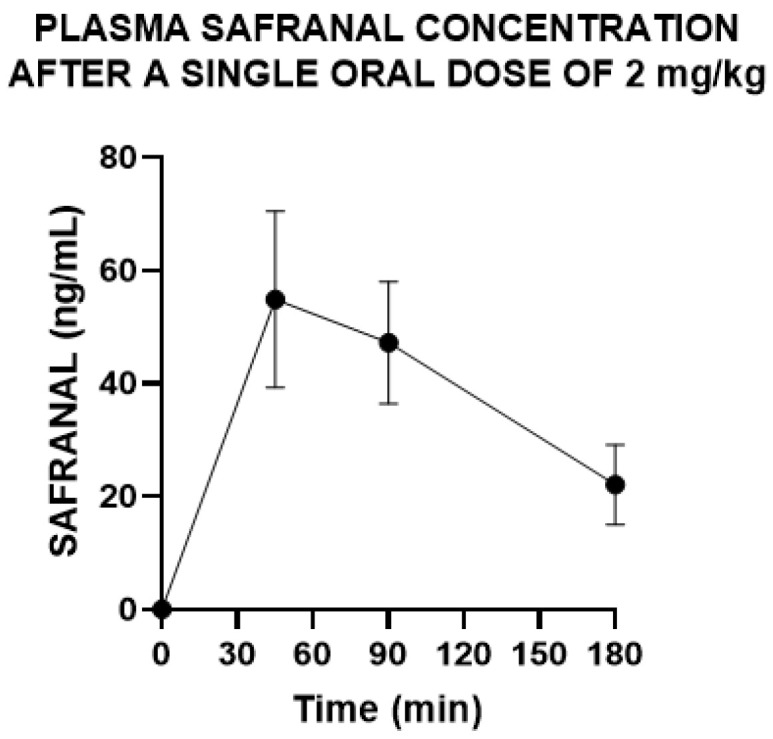
Plasma concentrations of safranal after oral administration of safranal-standardized saffron extract (SSE) 100 mg/kg (equivalent to 2 mg/kg of safranal). Data points are means ± standard error of the mean of 6 determinations.

Oral administration of SSE 100 mg/kg resulted in a time-limited rise in plasma safranal levels *F* (3, 20) = 29.81, *p* < 0.001, Figure 2. Post hoc analysis revealed that safranal rapidly rised in plasma at 45 min post-ingestion, keeping levels up to 90 min post extract ingestion and almost dissapearing at 180 min post SSE ingestion (*p* = 0.06).

### 3.2. Behavioral Actions of SSE

In order to evaluate the pharmacological actions of SSE on the behavior of aged mice, we selected three different behavioral tests related to the described actions of Saffron extracts: modulation of feeding behavior, anxiolysis in the open field and memory improvement in the NOR test. While feeding was evaluated throughout the treatment, anxiety and memory were evaluated at the end of the treatment with the SSE.

#### 3.2.1. Effects of SSE on Feeding Behavior and Body Weight Gain

**Figure 3:**
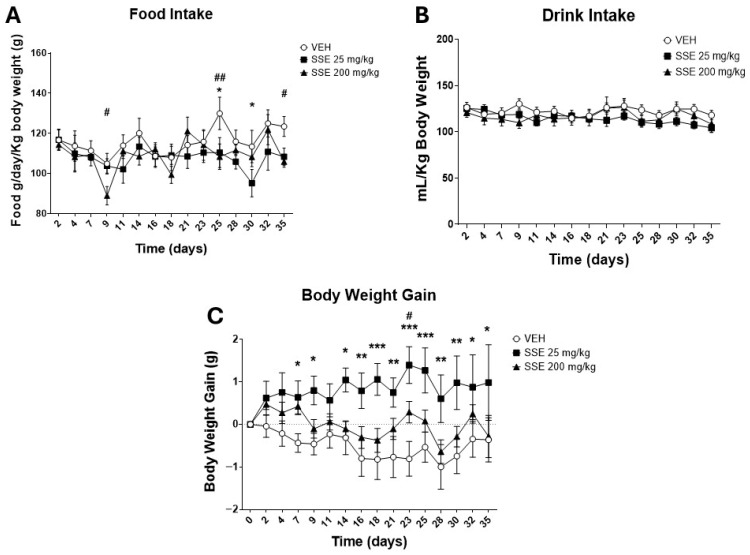
Effects of the administration of safranal-standardized saffron extract (SSE) (25 or 200 mg/kg/day in the drinking water) on (**A**) food intake, (**B**) water intake, and (**C**) cumulative body weight gain of aged (25-month-old) male mice. Data represents means ± SEM of 7–9 samples per group (VEH n = 9, SSE 25 n = 7; SSE 200 n = 8). Two-way ANOVA and Tukey’s test for multiple comparisons were performed: * = *p* < 0.05, ** = *p* < 0.01, and *** = *p* < 0.001 VEH vs. SSE 25 mg/kg group; # = *p* < 0.05, ## = *p* < 0.01 VEH vs. SSE 200 mg/kg group.

Treatment with SSE resulted in minor changes in feeding behavior that were day- (*F* (14, 315) = 22.30, *p* < 0.01) and dose- (*F* (2, 315) = 7.46, *p* < 0.001) dependent (Figure 3A). Thus, on certain days, especially at the end of the period of treatment, animals receiving SSE ate less than vehicle-treated ones, the dose of 200 mg/kg being the more effective one. In any case, the effects were of small intensity. Water intake (Figure 3A) was not affected by SSE treatment. Despite the minor effects on food intake, SSE 25 mg/kg, but not the 200 mg/kg dose, resulted in a clear improvement on body weight gain, antagonizing the slow decrease in body weight associated with aging (*F* (3, 336) = 22.30, *p* < 0.0001, Figure 3B).

#### 3.2.2. Effects of SSE on Anxiety-like Behaviors in the Open Field

**Figure 4:**
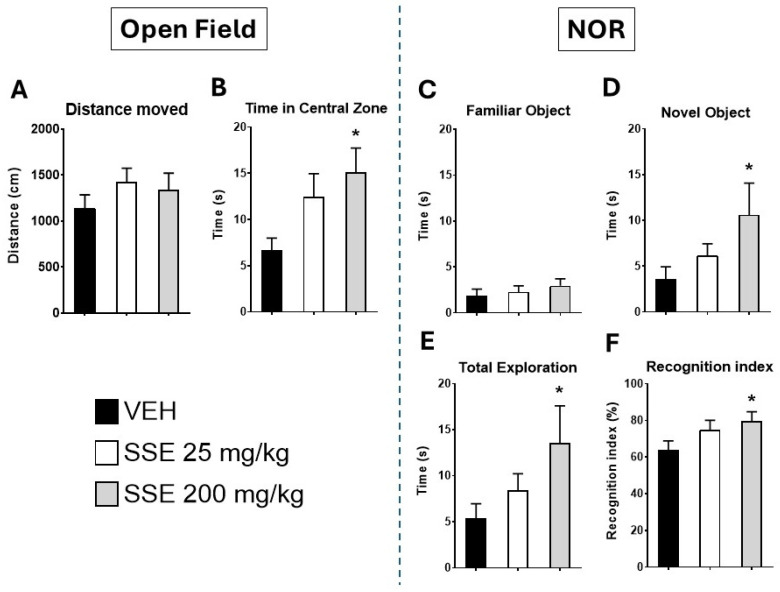
Effects of the administration of safranal-standardized saffron extract (SSE) (25 or 200 mg/kg/day for 35 days in the drinking water) on behavior displayed in the open field (left panel) and the novel object recognition test (NOR, right panel) by aged (25-month-old) male mice. (**A**) Total distance moved; (**B**) time spent in the central zone of the field; (**C**) time exploring the familiar object in the NOR test; (**D**) time exploring the novel object; (**E**) total exploration time; and (**F**) novelty recognition index. Data represents means ± SEM of 7–9 samples per group (VEH n = 9, SSE 25 n = 7; SSE 200 n = 8). One-way ANOVA and Dunnett’s test for comparisons with control group: * = *p* < 0.05 vs. VEH group.

To test motor activity and anxiety-like behaviors, we exposed aged animals treated with SSE to the open field. Animals treated with SSE did not exhibit alterations in motor behavior (Figure 4A; (*F* (2, 21) = 0.80, *p* = 0.46, not significant)). However, they spent more time in the center of the field, especially those treated with the 200 mg/kg dose (Figure 4B; (*F* (2, 21) = 3.80, *p* < 0.05)), a behavior indicative of anxiolysis.

#### 3.2.3. Effects of SSE on the Novel Object Recognition Memory Test

To investigate the effects of the SSE on memory in aged mice, we selected the NOR test. Animals receiving the 200 mg/kg of SSE explored the novel object for more time (*F* (2, 21) = 3.6, *p* < 0.05, Figure 4D) than vehicle-exposed animals, displayed higher exploration activity (*F* (2, 21) = 4.5, *p* < 0.05, Figure 4E), and had a greater novel object recognition index (*F* (2, 21) = 3.35, *p* < 0.05, Figure 4F), indicative of improvements in recognition memory.

### 3.3. Hypothalamic Expression of mRNA Gene Coding for Peptides Controlling Feeding After Treatment with SSE

**Figure 5:**
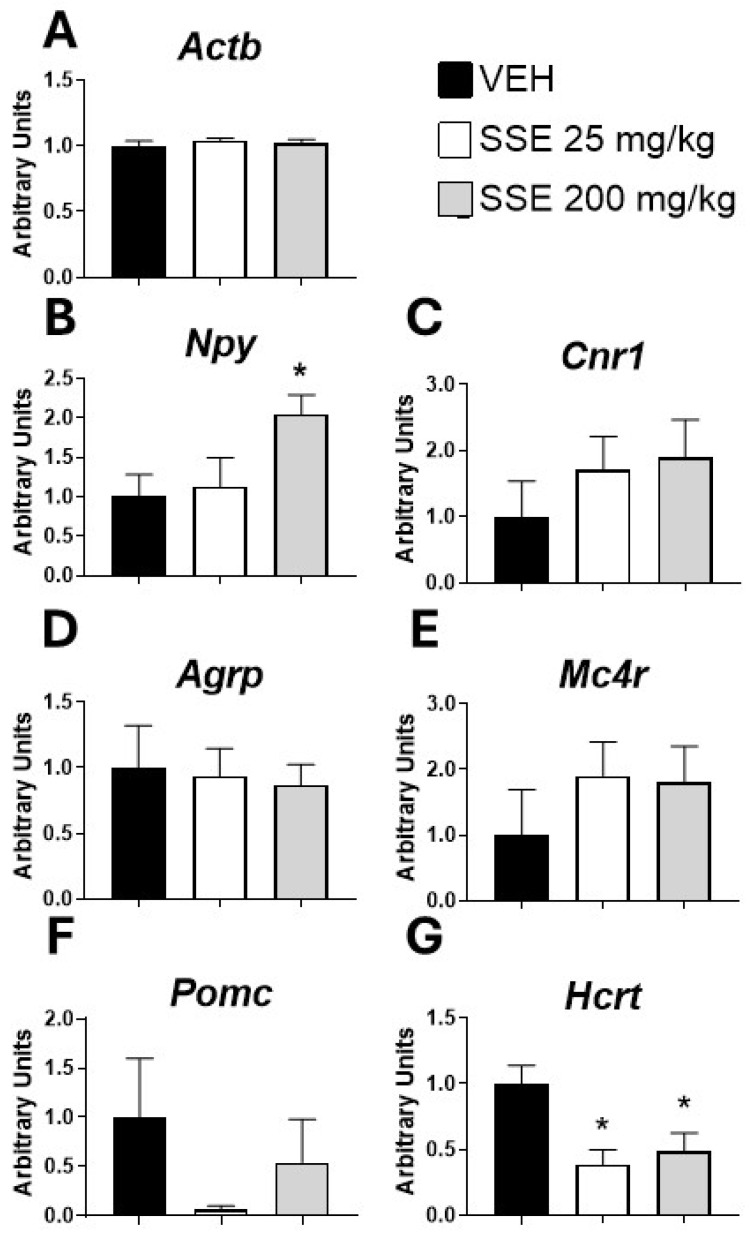
Real-time PCR measurement of mRNA expression in the brain hypothalamus of (**A**) Actin B (*Actb*), (**B**) *Npy*, (**C**) *Cnr1*, (**D**) *Agrp*, (**E**) *Mc4r*, (**F**) *Pomc*, and (**G**) *Hcrt* after sub-chronic treatment with safranal-standardized saffron extract (SSE) at doses of 25 or 200 mg/kg/day for 35 days in the drinking water. Histograms represent means ± SEM of 7–9 samples per group (VEH n = 9, SSE 25 n = 7; SSE 200 n = 8). One-way ANOVA and Dunnett’s test for post hoc comparisons with control group: * = *p* < 0.05 vs. VEH group.

Since previous reports have suggested that the active principles of saffron can modify feeding behavior and energy expenditure, we analyzed the hypothalamic expression of mRNA coding for main peptides controlling feeding (Figure 5). Results show that SSE treatment resulted in increased expression of the orexigenic *Npy* at the dose of 200 mg/kg (*F* (2, 20) = 3.53, *p* < 0.05, Figure 5B), as well as a decreased expression of *Hcrt*, a peptide coordinating energy expenditure and activating arousal and stress responses, at both doses (*F* (2, 21) = 6.36, *p* < 0.01, Figure 5G). Neither *Cnr1*, *Agrp*, *Mc4r*, nor *Pomc* mRNA expression was affected by the treatment (Figure 5C, Figure 5D, Figure 5E and Figure 5F, respectively). These results indicate a contribution of both *Npy* and *Hcrt* to the observed increase in weight gain and reduced anxiety-like behaviors.

### 3.4. Expression of mRNA Genes Involved in Emotional Control in the Amygdala of Animals Treated with SSE

**Figure 6:**
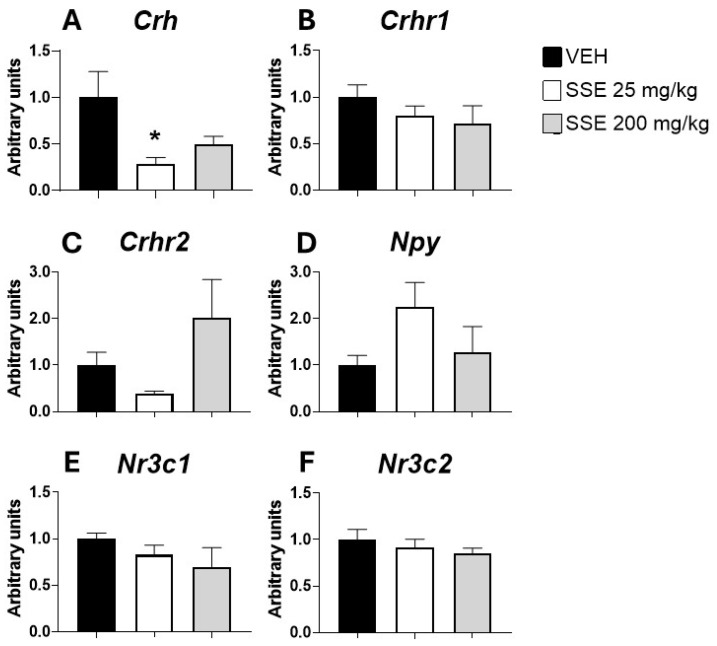
Real-time PCR measurement of mRNA expression in the brain amygdala of (**A**) *Crh*, (**B**) *Crhr1*, (**C**) *Crhr2*, (**D**) *Npy*, (**E**) *Nr3c1*, and (**F**) *Nr3c2* after sub-chronic treatment with safranal-standardized saffron extract (SSE) at doses of 25 or 200 mg/kg/day for 35 days in the drinking water. Histograms represent means ± SEM of 7–9 samples per group (VEH n = 9, SSE 25 n = 7; SSE 200 n = 8). One-way ANOVA and Dunnett’s test for post hoc comparisons with control group: * = *p* < 0.05 vs. VEH group.

Saffron has been traditionally used as a natural anxiolytic. Our behavioral data suggest that in aged mice, this effect is observed with the highest dose of the extract used. To further investigate the neurobiological mechanisms associated with this behavioral response, we evaluated the impact of the treatment on the mRNA expression of genes involved in emotional control in the brain amygdala (Figure 6). The data show that SSE treatment resulted in decreased expression of *Crh* (*F* (2, 21) = 3.69, *p* < 0.05, Figure 6A), a major pro-anxiogenic signal in the amygdala, and a non-significant trend to exhibit enhanced expression of *Npy* (*p* = 0.07, not significant, Figure 6D). This finding may suggest that the anxiolytic response observed after SSE is mediated by reducing *Crf* peptide expression in the amygdala. Neither *Crhr1*, *Crhr2*, *Nr3c1*, nor *Nr3c2* expression was affected by the treatment, although *Crhr2* also exhibited a non-significant trend to display increased levels of expression (*p* < 0.1), see Figure 6B, Figure 6C, Figure 6E and Figure 6F, respectively.

### 3.5. Expression of Proteins Involved in Neuroinflammation in the Prefrontal Cortex of Aged Mice Treated with SSE

**Figure 7:**
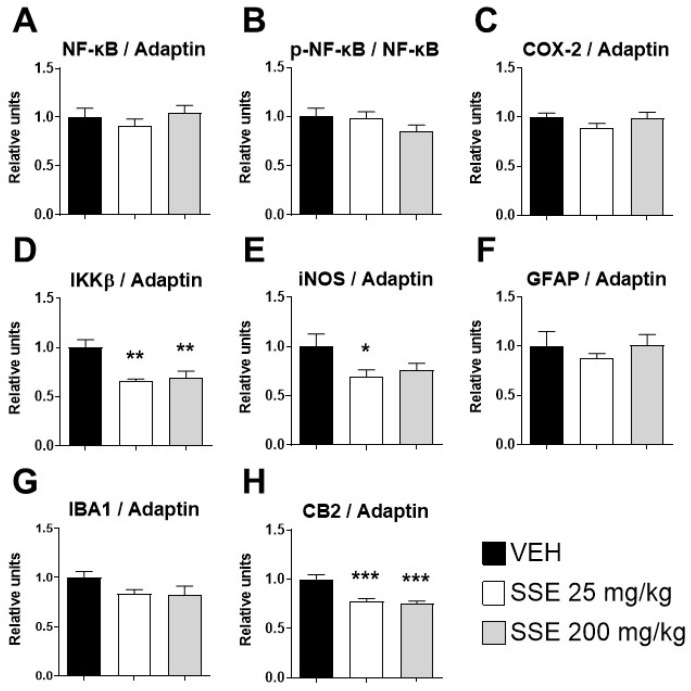

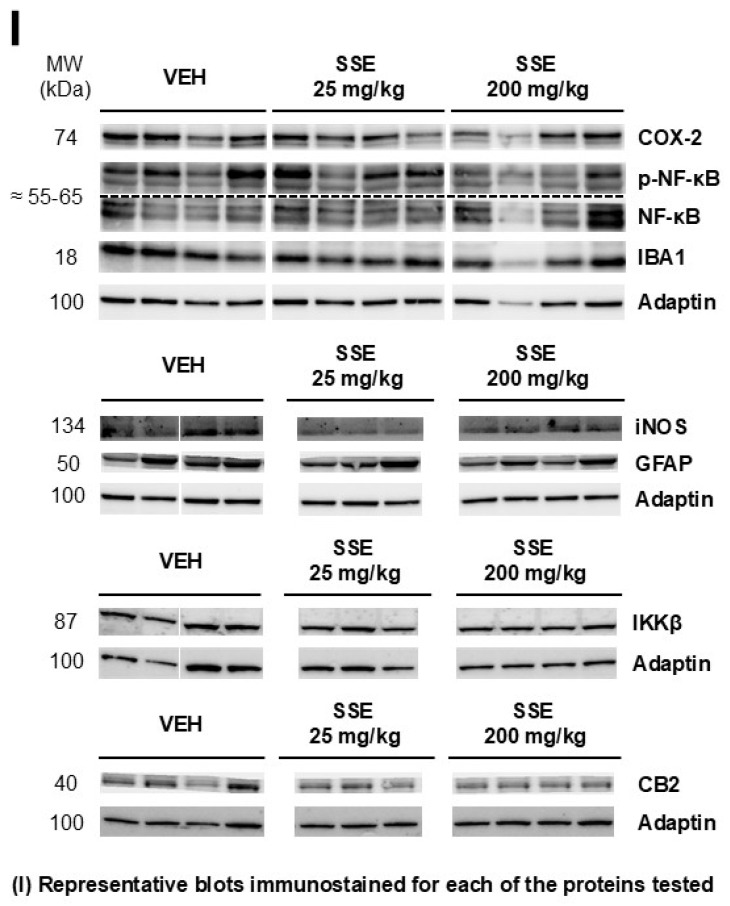
Analysis by Western blot of the protein expression of genes related to neuroinflammation in the prefrontal cortex of aged animals treated with safranal-standardized saffron extract (SSE). (**A**) NF-κB (Nuclear Factor kappa-light-chain-enhancer of activated B cells), (**B**) *p*-NF-κB (phosphorylated Nuclear Factor kappa-light-chain-enhancer of activated B cells), (**C**) COX-2 (Cyclooxygenase-2), (**D**) IKKβ (Inhibitor of Nuclear Factor kappa-B Kinase subunit beta), (**E**) iNOS (Inducible Nitric Oxide Synthase), (**F**) GFAP (Glial Fibrillary Acidic Protein), (**G**) IBA1 (Ionized Calcium-Binding Adapter Molecule 1), and (**H**) CB2 (Cannabinoid Receptor 2) after sub-chronic treatment with normalized saffron extract at doses of 25 or 200 mg/kg/day for 35 days in the drinking water. (**I**) Representative gels immunostained for each of the described proteins and for the reference protein Adaptin. Histograms represent means ± SEM of 7–9 samples per group (VEH n = 9, SSE 25 n = 7; SSE 200 n = 8). One-way ANOVA and Dunnett’s test for post hoc comparisons with control group: * = *p* < 0.05, ** = *p* < 0.01 and *** = *p* < 0.001 vs. VEH group. Dotted line indicates separation of NFkB from its phosphorylated form.

As described in the introduction, saffron has been associated with anti-inflammatory properties. Since neuroinflammation is a key ellement of synaptic plasticity and it has been associated with mood disorders and cognitive impairment in the aging process, we analyzed by, Western blot, whether the chronic administration of SSE modifies the protein expression of the inflammatory NFκB signaling cascade in the prefrontal cortex (see Figure 7). This signaling cascade is essential for the action of cytokines and chemokines signals activating neuroinflammation. SSE administration did not affect the protein expression of NFκB, its phosphorylated form, or that of COX-2 (Figure 7A–C). However, it reduced the expression of the main activatory protein of the NFκB cascade, IKKβ (*F* (2, 20) = 7.59, *p* < 0.01, Figure 7D), suggesting an inhibition of the signaling cascade that is confirmed by the reduced expression of target proteins, such as iNOS (*F* (2, 20) = 4.06, *p* < 0.05, Figure 7E) and CB2 receptors (*F* (2, 20) = 14.01, *p* < 0.001, Figure 7H). There were no overt effects on the expression of glial markers, such as the microglial marker IBA-1 (Figure 7F) or the astrocyte marker GFAP (Figure 7G). Overall, these results suggest an anti-inflammatory effect of the SSE on the prefrontal cortex, contributing to the beneficial effects observed on mood and memory.

### 3.6. Expression of mRNA Genes Involved in Memory Processes Control in the Hippocampus of Aged Mice Treated with SSE

**Figure 8:**
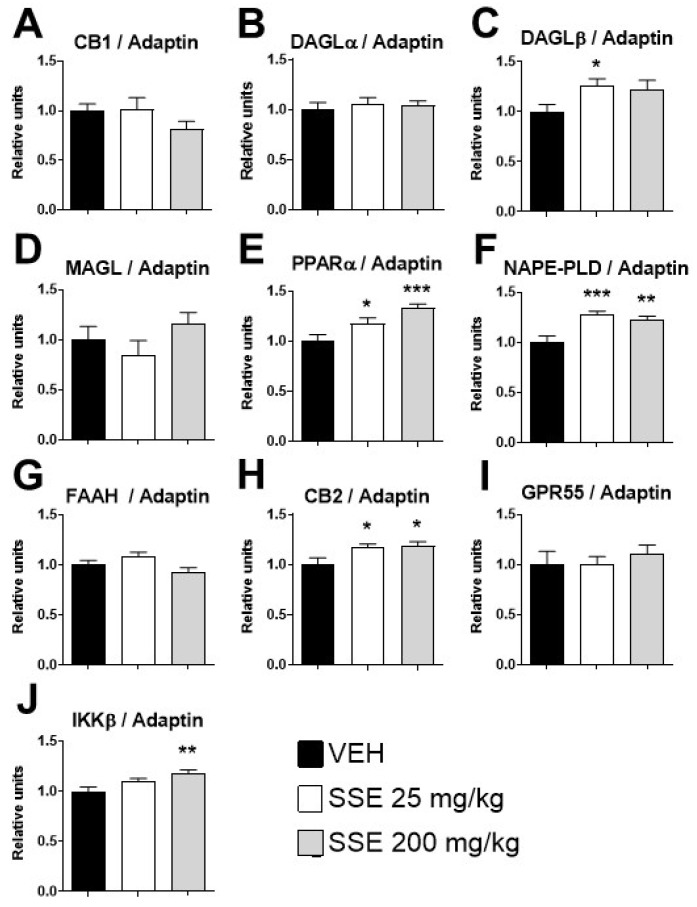

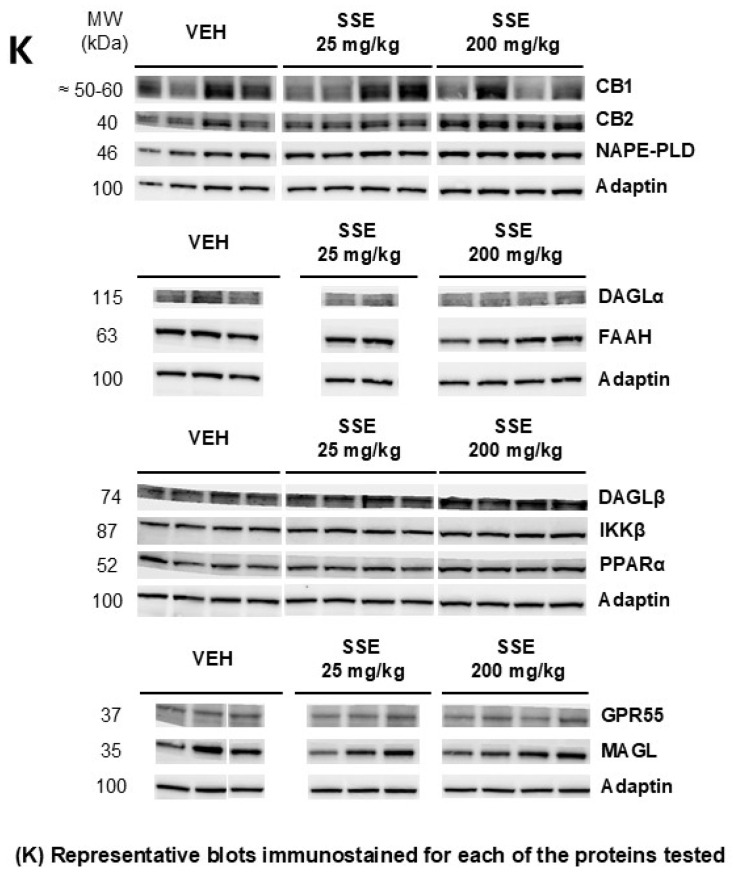
Analysis by Western blot of the protein expression of genes related to the endocannabinoid/paracannabinoid signaling system in the hippocampus of aged animals after sub-chronic treatment with safranal-standardized saffron extract (SSE) at doses of 25 or 200 mg/kg/day for 35 days in the drinking water: (**A**) CB1 (Cannabinoid Receptor 1), (**B**) DAGLα (Diacylglycerol Lipase Alpha), (**C**) DAGLβ (Diacylglycerol Lipase Beta), (**D**) MAGL (Monoacylglycerol Lipase), (**E**) PPARα (Peroxisome Proliferator-Activated Receptor Alpha), (**F**) NAPE-PLD (N-acyl Phosphatidylethanolamine-Specific Phospholipase D), (**G**) FAAH (Fatty Acid Amide Hydrolase), (**H**) CB2 (Cannabinoid Receptor 2), (**I**) GPR55 (G Protein-Coupled Receptor 55), and (**J**) IKKβ (Inhibitor of Nuclear Factor kappa-B Kinase subunit beta). (**K**) Representative gels immunostained for each of the described proteins and for the reference protein Adaptin. Histograms represent means ± SEM of 7–9 samples per group (VEH n = 9, SSE 25 n = 7; SSE 200 n = 8). One-way ANOVA and Dunnett’s test for post hoc comparisons with control group: * = *p* < 0.05, ** = *p* < 0.01 and *** = *p* < 0.001 vs. VEH group.

Our behavioral data suggest that SSE might improve cognition in aged animals, as demonstrated by enhanced recognition memory, which has a hippocampal-dependent basis. To this end, we measured the mRNA expression of *Bdnf*, its receptor *Ntrk2*, *Npy*, the glutamate receptors subunits *Gria1*, *Gria2*, *Grin1*, *Grin2a*, and *Grin2b*, and the glutamate metabotropic receptor, *Grm5*. All of them were affected by SSE administration (see Supplementary Figure S2). To extend the search for factors involved in plasticity at the hippocampal level, we analyzed the protein expression of the endocannabinoid/paracannabinoid signaling system, also involved in learning and memory (see Figure 8). The results showed that SSE barely affects the pathway of 2-AG signaling, a major memory regulator. Neither cannabinoid CB-1 receptor (Figure 8A), DAGLα (the major biosynthetic enzyme for 2-AG, Figure 8B), nor MAGL (main enzyme degrading 2-AG, Figure 8D) were affected by SSE. Only DAGLβ (an isoform present in glial cells that generates 2-AG) was increased after SSE administration (*F* (2, 21) = 3.8, *p* < 0.05, Figure 8C). However, SSE administration markedly affected the acylethanolamide pathway. The extract’s administration markedly increased both NAPE-PLD, the enzyme producing acylethanolamides (*F* (2, 21) = 8.94, *p* < 0.005, Figure 8E), and PPARα, a receptor for mono- and unsaturated anti-inflammatory acylethanolamides (*F* (2, 21) = 9.2, *p* < 0.005, Figure 8F). The protein expression of FAAH, the main enzyme degrading acylethanolamides, was not affected by the SSE (*F* (2, 21) = 3.8, *p* < 0.05, Figure 8G). These changes were associated with an increase in CB2 cannabinoid expression (*F* (2, 21) = 4.1, *p* < 0.05, Figure 8H), but not GPR55 (Figure 8I), two receptors involved in endocannabinoid signaling. In order to further investigate the inflammatory status of the hippocampus, we also examined the whole NFκB signaling pathway and regulated proteins. We only observed an increase in the expression of IKKβ (*F* (2, 21) = 6.7, *p* < 0.01, Figure 8I). Neither NFκB, its phosphorylated form, nor NFκB regulated pro-inflammatory proteins such as COX-2 o iNOS; glial markers (IBA-1 and GFAP) were not affected.

### 3.7. Expression of mRNA Genes Involved in Lipid Metabolism of Aged Animals Treated with SSE

**Figure 9:**
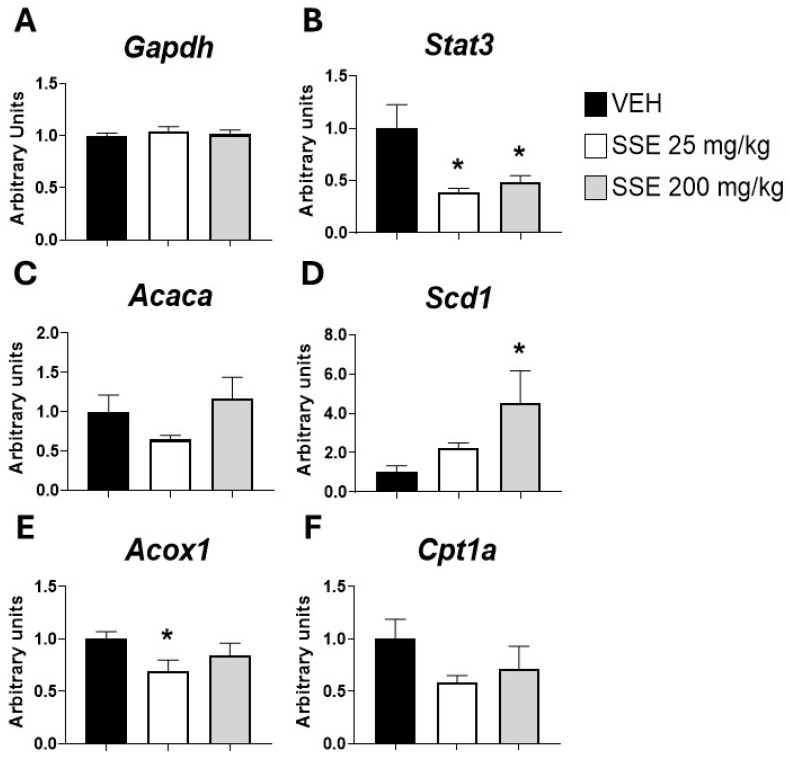
Real-time PCR measurement of mRNA expression in the liver of (**A**) *Gapdh* (Glyceraldehyde-3-phosphate dehydrogenase), (**B**) *Stat3* (Signal Transducer and Activator of Transcription 3), (**C**) *Acaca* (Acetyl-CoA Carboxylase Alpha), (**D**) *Scd1* (Stearoyl-CoA Desaturase 1), (**E**) *Acox1* (Acyl-CoA Oxidase 1), and (**F**) *Cpt1a* (Carnitine Palmitoyltransferase 1A) after sub-chronic treatment with safranal-standardized saffron extract (SSE) at doses of 25 or 200 mg/kg/day for 35 days in the drinking water. Histograms represent means ± SEM of 7–9 samples per group (VEH n = 9, SSE 25 n = 7; SSE 200 n = 8). One-way ANOVA and Dunnett’s test for post hoc comparisons with control group: * = *p* < 0.05 vs. VEH group.

Since we observed that animals treated with the low dose of SSE did not show a reduction but rather an increase in weight gain (Figure 3B), we analyzed whether the SSE might be modifying the mRNA expression of lipid metabolism-related genes in the liver. Data showed that *SSE* administration reduced *Stat3t*, the metabolic sensor controlling lipid biosynthesis (*F* (2, 18) = 3.93, *p* < 0.05, Figure 9B). The main fatty acid biosynthetic enzyme, *Acaca*, was not affected, but the key enzyme responsible for unsaturated fatty acid biosynthesis, *Scd1*, was increased (*F* (2, 21) = 3.5, *p* < 0.05, Figure 9D). These two effects were associated with a reduction in the expression of *Acox*, a gene related to fatty acid oxidation (*F* (2, 20) = 3.8, *p* < 0.05, Figure 9E), and a trend toward reduced *Cpt1a*, a carrier of fatty acids into the mitochondria (*F* (2, 18) = 2.2, *p* < 0.1, Figure 9F).

## 4. Discussion

Saffron offers a compelling case study of an ethnobotanical that has progressed from traditional use to modern pharmacological investigation. Its multimodal bioactivity is particularly relevant to aging, where complex and overlapping pathophysiological mechanisms demand broad interventions. The present study, using a safranal/crocin-standardized extract, demonstrates the pharmacological activity of testing extracts with known, balanced levels of safranal and crocins, thereby ensuring engagement of both central and peripheral targets. This is a first step towards the understanding of the pharmacology of complex formulations where PK diversity and target heterogeneity might lead to positive PD interactions, which are difficult to reproduce using isolated bioactive compounds [33]. The results provide evidence for the potential biomedical applications of saffron nutraceuticals beyond the providence stemming from its frequent adulteration.

As a proof-of-concept assessment of bioavailability, we first quantified safranal absorption following oral administration. We selected safranal because it is a chemical of saffron, whereas crocins are present even more abundantly in other plant extracts (e.g., *Gardenia* species) [34]. In male Wistar rats, oral administration of SSE produced a transient rise in plasma’s safranal, with concentrations peaking at ~45 min, remaining elevated to ~90 min, and approaching baseline by 180 min. This kinetic profile is consistent with published pharmacokinetics of safranal—an α,β-unsaturated aldehyde with high membrane permeability but limited stability in biological matrices and extensive first-pass metabolism—yielding rapid absorption followed by a swift decline in systemic levels [35]. Functionally, pharmacodynamic effects attributed to safranal—particularly in the CNS—are likely to depend on precise timing relative to this early peak and may involve contributions from rapidly formed metabolites alongside the transient parent compound [35]. Further studies should examine how repeated or continuous dietary administration of safranal influences plasma exposure and the formation of its metabolites.

Once we demonstrated bioactive levels of safranal in the plasma of animals after oral administration, we focused on the sub-chronic pharmacological effects of this SSE in aged animals. The administration this saffron for 35 days produced dose-dependent behavioral and metabolic responses in 2-year-old male mice. The low dose (25 mg/kg) prevented the body weight loss observed in the controls, while the high dose (200 mg/kg) was less effective. These findings suggest that the lower dose may have favored either energy expenditure saving (through a reduction in *Hcrt* expression [36,37]) or orexigenic signaling without triggering strong homeostatic compensation. This weight loss prevention effect is interesting and resembles what has been observed in stress-induced anorexia when an aqueous saffron extract is used at similar doses [38], an effect that opposes the induction of weight loss when saffron extracts are given under obesogenic conditions [39]. It is possible that the overall reduction in food intake, energy expenditure, and fat accumulation associated with aging can be assimilated at physiological levels with stress-induced anorexia, favoring the context-dependent orexigenic profile of SSE observed. This interpretation aligns with the effects of SSE in the liver, which shift lipid metabolism to reduced oxidation and increased biosynthesis. It is also supported by the expression of *Npy* mRNA—an orexigenic and anxiolytic signal—in the hypothalamus [40,41] observed at 200 mg/kg, although weight gain was evident only at 25 mg/kg. It is also congruent with the reduction *Hcrt* (orexin and hypocretin) expression that, despite being a pro-orexigenic signal [42], also promotes stress, increased arousal, and enhanced energy expenditure [36,37,43]. These results also suggest that SSE activity depends on the interaction with stress responses and mood. Further research is needed to understand the metabolic effects of large doses of saffron constituents, focusing on how the context (obesogenic diets, stress situation, and age, etc., …) promotes or reverses satiety or modulates energy expenditure at different doses.

Regarding the anxiolytic actions of SSE, from an emotional standpoint, anxiolytic-like behavior in the open field was observed at 200 mg/kg, without being associated with motor hyperactivity, indicating a genuine anxiolytic effect rather than psychostimulation. This effect correlates with reduced hypothalamic *Hcrt* expression, a neuropeptide linked to arousal and stress [36,37], and with an increased hypothalamic *Npy,* which exerts both orexigenic and anxiolytic actions [40,41]. Supporting this hypothesis, we found that in the amygdala, a critical hub for stress and anxiety, SSE administration resulted in a reduction in *Crh* expression (significant at 25 mg/kg and trending at 200 mg/kg). These molecular effects converge on the modulation of the hypothalamic–pituitary–adrenal (HPA) axis, reducing stress while favoring *Npy*. Since *Crh* is a major signal activating stress responses, anxiety, and arousal [44], the observed reduction in its expression further supports the anxiolytic actions of SSE.

In addition to the effects on the amygdala, we explored the potential effects of SSE on neuroinflammation signatures, a process associated with mood alterations and anxiety [45], especially in the prefrontal cortex (PFC), and with memory consolidation in the hippocampus [46]. The PFC is a crucial hub for emotions and working memory, and its dysfunction is linked with major mood disorders, which can impact cognition [47,48]. In the animals exposed to SSE, the PFC exhibited a downregulation of CB2 and IKKβ protein with both doses, and a reduction in iNOS at 25 mg/kg, without changes in NF-κB or glial markers (GFAP and IBA1). Since IKKβ is a major regulatory protein activating NF-κB signaling, the reduction in IKKβ protein suggests a mild anti-inflammatory effect, targeting downstream-located elements of inflammatory cascades, including iNOS and CB2, without triggering broad glial remodeling. Conversely, in the hippocampus, the 25 mg/kg dose increased IKKβ, a paradoxical effect possibly reflecting a localized adaptive response linked to metabolic signaling or compensatory processes during synaptic remodeling and probably facilitating synaptic plasticity and learning [49]. Again, the complex PK-PD interactions of the main constituents of the extract are likely relevant for understanding the different dose- and brain area-dependent pharmacological actions of SSE observed, a problem that demands further research.

The memory improvement in the novel object recognition test at 200 mg/kg aligns with enhanced cognitive performance reported for saffron constituents in both preclinical and clinical settings [19,20]. This effect was not accompanied by changes in BDNF, TRKB, or glutamate receptor expression, suggesting alternative pathways, such as that of the endocannabinoid/paracannabinoid system. Regarding this hypothesis, data showed that the hippocampus exhibited clear changes in endocannabinoid-related pathways, which are highly relevant for memory and synaptic plasticity. While CB1 receptor expression remained unchanged, CB2 protein was increased (especially at 25 mg/kg), accompanied by elevated DAGLb expression, suggesting enhanced 2-arachidonoylglycerol (2-AG) synthesis. CB2 is often associated with neuroimmune modulation and synaptic homeostasis rather than classic psychotropic effects mediated by CB1 [50,51], further supporting the IKKβ actions described above. Additionally, the paracannabinoid system (monounsaturated acylethanolamides/PPARα pathway) was upregulated, as indicated by increased NAPE-PLD and PPARα expression, without changes in FAAH. This is critical, since the bioactive lipid OEA synthesized by NAPE-PLD from membrane phospholipids acts through PPARα receptors to support memory and metabolic homeostasis. The activation of the OEA-PPARα axis facilitates lipid utilization and anti-inflammatory signaling, contributing to memory consolidation [52].

Overall, the present brain results indicate that SSE exerts an important modulatory effect on molecular mechanisms involved in the functional integration of the interconnected PFC–amygdala–hippocampus–hypothalamus network. This complex system acts in concert to maintain emotional homeostasis, memory, feeding behavior, energy expenditure, and sleep [53,54]. The observed molecular changes in the prefrontal cortex, amygdala, and hippocampus are consistent with established models of prefrontal–limbic regulation of emotional memory, particularly involving PFC projections to the basolateral amygdala and hippocampus, and warrant further investigation to better understand the mechanisms underlying SSE activity. Such studies should include an assessment of the ESR2/BDNF axis, which regulates both memory and mood, especially in the context of aging [55,56]. Estrogen receptor β is abundantly expressed in these regions and has been shown to modulate affective behavior and synaptic plasticity, in part through regulation of BDNF signaling as well as the NPY/CRH balance. Characterizing changes in this axis would provide further support for enhanced plasticity within the PFC–amygdala–hippocampal circuitry that underlies memory and emotional regulation. This will be particularly relevant if demonstrated in female subjects, given the age-associated decline in estradiol following menopause [57], as well as in males, in whom aging is also associated with progressive reductions in estradiol and testosterone production [58].

Finally, we examined the liver, since along with aging, important metabolic adaptations occur, contributing to fat deposition, dysregulation of lipid homeostasis, and insulin resistance. All these factors also contribute to brain alterations, impacting both mood and cognition [59]. In the liver, both doses downregulated STAT3, a key regulator of energy homeostasis and leptin signaling [60]. At 25 mg/kg, ACOX (involved in peroxisomal β-oxidation) decreased, while CPT1 tended to be lower, suggesting reduced fatty acid oxidation. This aligns with the observed weight gain at the lower dose. Conversely, 200 mg/kg increased SCD1 expression, favoring lipogenesis, though without overt weight gain, possibly due to central or peripheral energy partitioning effects mediated by PPARα and endocannabinoid pathways [61,62]. These data underscore a bidirectional brain–liver interaction, where saffron constituents modulate not only central neuropeptide tone but also peripheral lipid metabolism, shaping energy balance.

## 5. Conclusions and Limitations

Taken together, these results highlight a dose-dependent neuro-metabolic modulation by SSE, involving the following:

- Low dose (25 mg/kg): Favoring weight gain and mild anti-inflammatory responses (PFC) with selective hippocampal activation of endocannabinoid/paracannabinoid signaling and increased IKKβ.
- High dose (200 mg/kg): Promoting anxiolytic behavior and memory enhancement, linked to endocannabinoid and acylethanolamide–PPARα signaling in the hippocampus, and a decrease in hypothalamic *Hcrt* expression.

These effects likely result from a synergistic interaction between safranal, crocins, and their metabolites, influencing central neuropeptide systems (*Npy* and *Crh*), endocannabinoid and PPAR pathways, and hepatic lipid metabolism, which together shape emotional regulation, cognitive performance, and energy homeostasis in aging organisms. In this regard, we make the following clinically oriented conclusions:

- This study suggests that the use of a standardized saffron extract similar to that presented here can be translated to humans, as the dosage employed falls within the range equivalent to that used in traditional practices and clinical trials in humans.
- Second, we emphasize the importance of focusing on aged populations, in whom improvements in quality of life may be particularly meaningful due to the accumulation of symptoms, such as memory decline, mood alterations, and sleep disturbances. In this context, we also stress the need to include studies in postmenopausal/aged women, given the clear sex-associated increase in these symptoms following estradiol decline.

Several limitations must be considered in the present study. First, this is an exploratory study using a complex water-based SSE standardized for safranal content. There is a clear need to establish chemical fingerprint reproducibility, namely, a standardized MS-based method for content analysis and plasma detection of the main constituents. Second, inter-individual variability in phytochemical metabolism must be considered, as it may limit efficacy. Third, the contribution of each active ingredient in the extract to the overall pharmacological activity needs to be confirmed using individual purified components (e.g., safranal, crocins, or its bioavailable derivative, crocetin) to identify mechanisms of action and potential interactions among saffron constituents. Fourth, anxiety can be a confounding factor in cognitive studies, with the influence of sex, type of test, and age needing to be controlled to understand the type of cognitive benefits derived upon saffron extract ingestion. Lastly, the study was conducted in aged males only; therefore, it should be extended to aged females before any generalization of saffron’s effects as a nutritional supplement for healthy aging can be made. In fact, this is a crucial step in saffron research, given the well-established association between estradiol, ESR2/BDNF signaling, neuroprotection, and appropriate memory and mood outcomes. This consideration is also relevant in males, as brain estradiol is largely derived from the aromatization of testosterone, which itself declines with age.

## Data Availability

A detailed protocol outlining the research question, key design features, and analysis plan was prepared before the commencement of the study. This protocol was not registered in any public repository. This published article and its Supplementary Information files include all data generated or analyzed during this study. Raw data are available on reasonable request to the corresponding author.

## Supplementary Materials

The following supporting information can be downloaded at: https://www.mdpi.com/article/10.3390/nu18020291/s1, Table S1: Primer references for qPCR; Table S2: Primary antibodies used for protein expression by Western blotting; Method S1: Bioanalytical HPLC-UV method for the analysis of contents of safranal and crocins in the SSE; Figure S1: representative chromatogram of a sample of SSE; Figure S2: real-time PCR analysis of mRNA expression of genes involved in memory formation.
