# Supplementary material for: Safranal-Standardized Saffron Extract Improves Metabolic, Cognitive, and Anxiolytic Outcomes in Aged Mice via Hypothalamic–Amygdalar Peptide Modulation
Source: Nutrients. 2026 Jan 16;18(2):291. doi: 10.3390/nu18020291 (PMC12845050; doi:10.3390/nu18020291)

SUPPLEMENTARY MATERIALS

Metabolic, Cognitive, and Anxiolytic Benefits of a Safranal-Standardized Saffron Extract in Aged Male Mice via Remodeling of Hypothalamic–Amygdalar NPY/CRH Expression

Juan A. Navarro[1,2], Ana Gavito[1,3], Sonia Rivas[1], Alonso Rodríguez-Martín[1], Elena Baixeras[4], J. Decara[1,3], Pedro J. Serrano-Castro[2,5], Yolanda Alfonso[6], Carlos Sanjuan[6], Antonia Serrano[1,3], Fernando Rodríguez de Fonseca [1,2,5,*]

1. **TABLES OF PRIMERS AND ANTIBODIES**

2. **METHOD FOR ANALYSIS OF SAFRANAL/CROCINS CONTENT**

3. **PCR DATA IN THE HIPPOCAMPUS**

Supplementary Table S1. Primer references for TaqMan® Gene Expression Assays. The nomenclature, gene name, assay identifier, GenBank accession number, and amplicon length (in base pairs) of each analyzed gene are provided.

Gene nomenclature	Gene name	Assay ID	Nº GenBank	Amplicon length (bp)
Actb	beta-Actin	Mm02619580_g1	AK075973.1	143
Gapdh	Glyceraldehyde-3-phosphate dehydrogenase	Mm99999915_g1	NM_001289726.1	107
Npy	Neuropeptide Y	Mm01410146_m1	NM_023456.3	130
Cnr1	Cannabinoid receptor 1	Mm00432621_s1	NM_007726.5	69
Agrp	Agouti related neuropeptide	Mm00475829_g1	NM_001271806.1	86
Mc4r	Melanocortin 4 receptor	Mm00457483_s1	NM_016977.4	79
Pomc	Pro-opiomelanocortin-alpha	Mm00435874_m1	NM_001278581.1	60
Hcrt	Hypocretin	Mm01964030_s1	NM_010410.2	168
Stat3	Signal transducer and activator of transcription 3	Mm01219775_m1	NM_011486.6	75
Acaca	Acetyl-Coenzyme A carboxylase	Mm01304285_m1	NM_133360.1	123
Scd1	Stearoyl-Coenzyme A desaturase 1	Mm00772290_m1	NM_009127.4	60
Acox1	Acyl-Coenzyme A oxidase 1	Mm00443575_g1	NM_001271898.2	101

Cpt1a	Carnitine palmitoyltransferase 1a	Mm01231183_m1	NM_013495.2	60
Crh	Corticotropin releasing hormone	Mm01293920_s1	NM_205769.2	128
Crhr1	Corticotropin releasing hormone receptor 1	Mm00432670_m1	NM_007762.4	74
Crhr2	Corticotropin releasing hormone receptor 2	Mm00438308_m1	NM_001288618.1	73
Nr3c1	Nuclear receptor subfamily 3, group C, member 1	Mm00433832_m1	NM_008173.3	68
Nr3c2	Nuclear receptor subfamily 3, group C, member 2	Mm01241596_m1	NM_001083906.1	85
Bdnf	Brain derived neurotrophic factor	Mm04230607_s1	NM_001048139.1	92
Ntrk2	Neurotrophic tyrosine kinase, receptor, type 2	Mm00435422_m1	NM_001025074.3	92
Gria1	Glutamate receptor, ionotropic, AMPA1 (alpha 1)	Mm00433753_m1	NM_001113325.2	78
Gria2	Glutamate receptor, ionotropic, AMPA2 (alpha 2)	Mm00442822_m1	NM_001039195.1	67
Grin1	Glutamate receptor, ionotropic, NMDA1 (zeta 1)	Mm00433790_m1	NM_001177656.2	63
Grin2a	Glutamate receptor, ionotropic, NMDA2A (epsilon 1)	Mm00433802_m1	NM_008170.2	84
Grin2b	Glutamate receptor, ionotropic, NMDA2B (epsilon 2)	Mm00433820_m1	NM_008171.3	61
Grm5	Glutamate receptor, metabotropic 5	Mm00690332_m1	NM_001081414.2	97

Supplementary Table S2. Primary antibodies used for protein expression analysis by Western blotting. The antigen, manufacturing details, source, and dilution used for each antibody are provided.

Antigen	Manufacturing details	Source	Dilution
γ-Adaptin	BD Biosciences (#610385)	Mouse	1:2000
DAGLα	Biorbyt (#Orb156533)	Rabbit	1:100
DAGLβ	Biorbyt (#Orb182976)	Rabbit	1:100
CB1	Abcam (#ab23703)	Rabbit	1:200
CB2	Abcam (#ab3561)	Rabbit	1:200
FAAH	Cayman (#101600)	Rabbit	1:100
NAPE-PLD	Abcam (#Ab95397)	Rabbit	1:1000
IKKβ	Cell signaling (#2684S)	Rabbit	1:1000
PPARα	Abcam (#ab15270)	Rabbit	1:500
GPR55	Abcam (#ab203663)	Rabbit	1:500
MAGL	Abcam (#ab24701)	Rabbit	1:500
iNOS	R&D systems (#MAB9502)	Mouse	1:200
COX2	Cell signaling (#12282)	Rabbit	1:500
p-NF-κB (Ser536)	Cell signaling (#3033S)	Rabbit	1:1000
NF-κB	Cell signaling (#8242S)	Rabbit	1:1000
GFAP	Sigma-Aldrich (#G3893)	Mouse	1:400
IBA1	Abcam (#ab5076)	Rabbit	1:500

Materials and Methods for Saffron Extract Characterization

Chemicals and Reagents

Ultra-pure water (ISO type 1, Milli-Q system, Millipore, USA), HPLC-grade methanol, and HPLC-grade acetonitrile (Merck, Germany) were used for all chromatographic procedures. Reference standards included safranal (~75% purity), crocin I (99.1% purity), and crocin II (99.1% purity) (Sigma-Aldrich, USA). Determination of picocrocin and additional crocins was performed using crocin I as a reference marker.

Equipment

An ultrasonic shaker (P Selecta, Spain), vortex mixer, and precision balance (Mettler Toledo XS Excellence, Switzerland; d = 0.1 mg, precision ±0.0001 g) were used for sample preparation. Moisture content was measured with a moisture analyzer (Mettler Toledo HB43-S, Switzerland; range 100–160 °C, precision ±0.001 g). Chromatographic separation was carried out on an Agilent 1200 HPLC system (Agilent Technologies, USA) equipped with an isocratic pump, an autosampler, and a diode-array detector (DAD). The chromatographic column was a Eurospher RP C18 (250 × 4.6 mm, 5 µm; KNAUER, Germany) or equivalent.

Sample Preparation

Approximately 2.5 g of saffron extract were dried in a moisture analyzer in accordance with ISO 3632. A 50 mg portion of the dried extract was accurately weighed and dissolved in a 10 mL volumetric flask with methanol–water (50:50, v/v) to obtain a 5.0 mg/mL solution. The solution was vortex-mixed for 10 s and sonicated for 5 s, followed by filtration through an RP-C18 filter previously conditioned with methanol–water (50:50, v/v). Aliquots of 0.3 mL of the filtrate were transferred into HPLC vials for chromatographic analysis.

Preparation of Standards and Calibration Curve

Standard solutions of crocin I, crocin II, and safranal were prepared in methanol–water (50:50, v/v) at appropriate concentrations. The same procedure used for sample preparation (vortexing, sonication, and filtration) was applied to ensure consistency. Calibration curves were constructed by plotting peak area against known concentrations of each standard compound. Picocrocin and other crocins were quantified using the calibration factor derived from crocin I.

Chromatographic Conditions

Separation of crocins, picocrocin, and safranal was performed on the Agilent 1200 HPLC-DAD system. The column temperature was maintained at 20 °C, and the mobile phase consisted of a linear gradient of methanol (10–100%) in water containing 15% acetonitrile. The flow rate was 1.0 mL/min, with system pressure maintained below 4 MPa. Detection wavelengths were set at 440 nm for crocins, 250 nm for picocrocin, and 310 nm for safranal.

Retention times (mean ± SD) were as follows: picocrocin (10.693 ± 0.950 min), HTCC (16.826 ± 0.950 min), kaempferol (20.162 ± 0.950 min), crocin I (27.246 ± 0.950 min), crocin II (30.651 ± 0.950 min), crocin III (35.016 ± 0.950 min), crocin IV (38.228 ± 0.950 min), safranal (40.310 ± 0.950 min), crocin V (40.519 ± 0.950 min), and crocin VI (43.909 ± 0.950 min).

Quantification and Data Analysis

Concentrations of crocins, picocrocin, and safranal were calculated based on chromatographic peak areas relative to calibration standards. Quantitative results were expressed as percentages using the following formula:

% = [(Area_sample / (concentration × %DC)_sample) / (Area × %purity)_standard / (concentration × %DC × 100%)_standard] × 100

where DC represents the dilution correction factor.

References

The method was developed based on internal Euronutra Laboratory protocols and adapted from the published procedure in Economic and Qualitative Traits of Italian Alps Saffron.

SUPPLEMENTARY FIGURE S1:

Representative Chromatograph of Standardized Saffron Extract

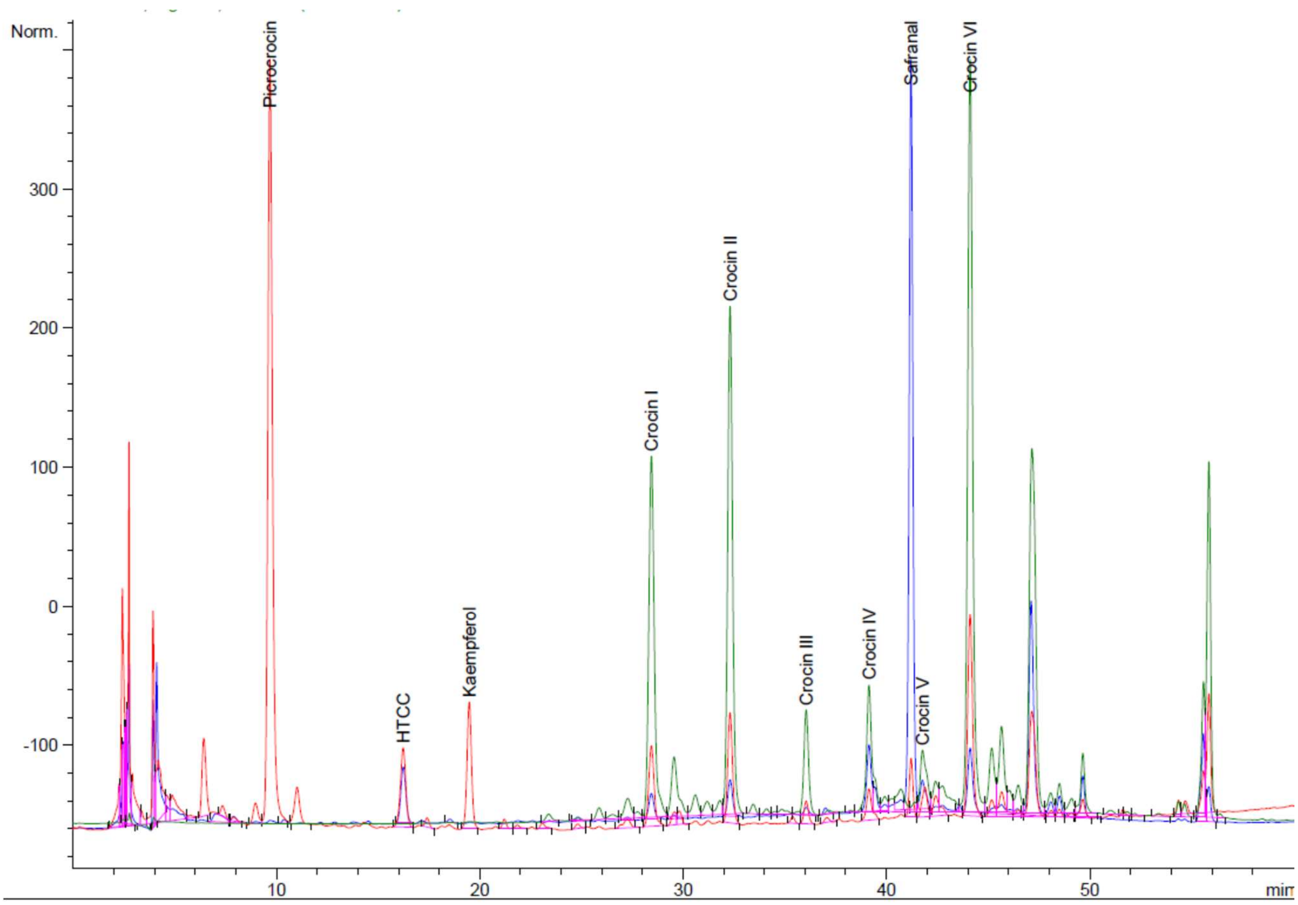

SUPPLEMENTARY FIGURE S2:

REAL-TIME PCR ANALYSIS OF mRNA EXPRESSION OF GENES ASSOCIATED WITH CONTROL OF MEMORY FORMATION IN THE HIPPOCAMPUS

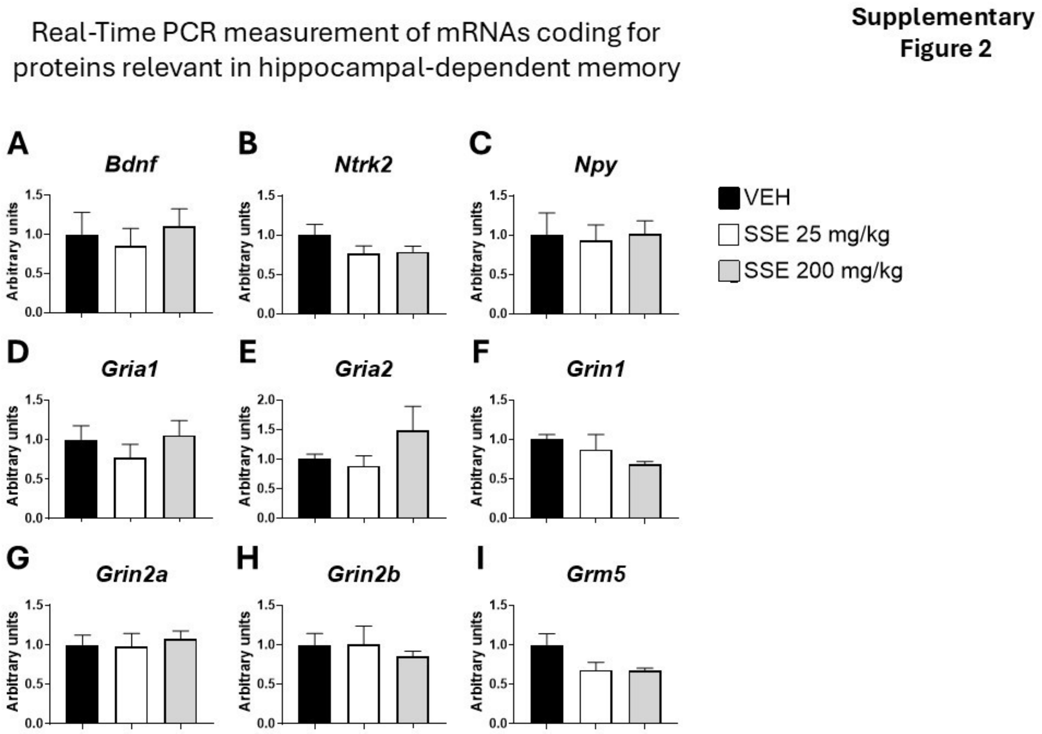

Supplement: Supplementary file 1 [file nutrients-18-00291-s001.zip › nutrients-4008324-supplementary.pdf]
